# S14-1 Sports promotion in young people: good practice interventions in selected European countries

**DOI:** 10.1093/eurpub/ckad133.067

**Published:** 2023-09-11

**Authors:** Chair(s):, Sonja Kahlmeier

**Affiliations:** The Swiss Distance University of Applied Sciences, Switzerland

## Abstract

**Purpose:**

Which flagship sport promotion interventions for young people exist in different European countries and what could other countries gain from knowing about them? This symposium gives an overview of effective sport promotion interventions in seven European countries and presents and discusses selected promising interventions and programmes.

**Symposium description:**

A research group identified effective sport promotion interventions in seven selected European countries with a potential to be transferred to Switzerland, including: Austria, Germany, England, Finland, France, The Netherlands, and Norway. National strategies with comprehensive and overarching measures or low-threshold interventions targeting less physically active subgroups in communities or schools have been identified as promising interventions. We will highlight and discuss selected examples in this symposium:

The national strategy “On the Move” from Finland promotes physical activity in different settings such as schools, families, and childcare. More than 90 per cent of all comprehensive schools in Finland are “Schools on the Move”. As a result, the school culture has become more physically active.

Community sport coaches in 98% of municipalities in the Netherlands provide physical activity education, organize events and activities, and advise clubs and childcare organizations. They work with local and social organizations.

Girls Active and Change4Life Sports Club from England are low-threshold programmes that promote sport at school, addressing all school aged children, including those who are inactive.

**Conclusions:**

This symposium will discuss benefits, barriers, and key aspects related to effectiveness of good practice interventions and identify the most important considerations that need to be addressed to transfer effective sport promotion policies or programmes from one country to another.

